# Primary care patient and practice member perspectives on weight loss medications: challenges and opportunities

**DOI:** 10.3389/fmed.2025.1584799

**Published:** 2025-07-07

**Authors:** Jodi Summers Holtrop, Caroline Tietbohl, Leigh Perreault, Lauri Connelly, Peter C. Smith, Johnny Williams

**Affiliations:** ^1^Department of Family Medicine, University of Colorado Anschutz Medical Campus, Aurora, CO, United States; ^2^Adult and Child Center for Outcomes Research and Delivery Science, University of Colorado Anschutz Medical Campus, Aurora, CO, United States; ^3^Division of Endocrinology, Metabolism and Diabetes, Department of Medicine, University of Colorado Anschutz Medical Campus, Aurora, CO, United States; ^4^Department of Epidemiology, Colorado School of Public Health, Aurora, CO, United States

**Keywords:** weight loss, medication, primary care, interviews, practice perspectives, patient perspectives

## Abstract

**Introduction:**

Obesity is a prevalent and concerning chronic condition; however, evidence-based interventions are available for treatment. With the arrival of newer and more effective anti-obesity medications, questions emerge regarding how these medicines can and should be used and how they affect the practice of primary care medicine. The objective of this study was to examine the many and intersecting factors affecting use and impact of these medicines.

**Methods:**

Qualitative study of interviews with primary care practice members and their patients in one Colorado health system (*n* = 56 practices) over 3 years. Thematic analysis was used to triangulate responses from patients and practice members.

**Results:**

Key themes from both practice members and patients were highly consistent revealing the following categories of benefits and burdens: (1) the new medicines are a “game changer” for practice and changed lives for patients, (2) there is significant burden for all in obtaining the medications for many patients, (3) not all patients should be on the medications, (4) the medications have changed the conceptualization of obesity for patients and providers, and practice teams, and (5) the availability of these medications have changed the practice of treating obesity in important ways. It was further identified that a cascade of events involving various factors with a “right fit” between the patient, provider, and other factors were needed to make way for access to and effective use of these medications.

**Discussion:**

The arrival of highly effective weight loss medications may invigorate efforts to integrate weight management into primary care, but the implications of this shift are still unknown. Further exploration of the long-term effects on patients, providers and care paradigms is warranted.

## Introduction

The obesity epidemic is one of the greatest health challenges of our time, affecting 42% of United States adults (1) and resulting in United States $1.4 trillion in annual health care costs (2). Obesity’s growing impact on health over the past four decades has been met with surprisingly few effective treatments that can be deployed at such a scale. While lifestyle modifications (i.e., diet, exercise and behavior change) remain foundational treatments for obesity, they typically result in modest weight loss, which is rarely maintained long term (3). In contrast, bariatric surgery boasts 46%–74% excess weight loss 10 years post-operatively (4), but is not scalable to a large population. Current literature indicates that only 12% of United States adults with a body mass index (BMI) > 25 kg/m^2^ receive adequate weight management care, as evidenced by the use of a weight-related ICD-10 code (5). Of those who did receive obesity care, approximately 95% were provided with lifestyle advice alone, highlighting the critical need to systematically address the barriers impeding effective weight management in primary care, including the use of anti-obesity medications (AOMs).

Anti-obesity medications have long-existed for addressing weight loss and popular options included drugs such as phentermine and orlistat. AOMs were first approved by the United States Food and Drug Administration in 1959. Since this time, history of the AOMs is rife with safety issues, limited efficacy and withdrawals from the market (6). Recently, a new classification of medications have emerged including glucagon-like peptide-1s (GLP-1s) and GLP-1/Glucose-dependent insulinotropic polypeptide (GIP) dual agonists. These new medications have the potential to close the gap between lifestyle modification alone and bariatric surgery. Semaglutide 2.4 mg (a GLP-1) has demonstrated an average weight loss of 15% weight loss over 68 weeks (7) and now includes an indication for the prevention of major adverse cardiovascular events in people with overweight or obesity without diabetes (8). Tirzepatide (a GLP-1/GIP) has demonstrated an even greater average of 22.5% weight loss at 72 weeks (9) and aims to reduce multi-morbidity, beyond cardiovascular events, in people with overweight or obesity without diabetes (10). Despite these promising results, neither medication has been widely adopted in routine medical practice, particularly in primary care (11). The removal of other promising AOMs from the market due to safety concerns (12) has resulted in significant hesitancy in prescribing. In one large cohort study, only 1.3% of patients with a BMI over 30 were prescribed any AOM, and the prescribing was done by a small minority of providers (13). The recent arrival of a new generation of highly effective medications to the United States market has renewed interest in the broader use of AOMs from both patients and providers.

Primary care is widely recognized as the cornerstone of the healthcare system, where patients receive assistance for preventive and chronic disease management, both of which often require lifestyle changes such as diet and exercise. However, many primary care practices encounter substantial barriers to delivering effective weight management. The introduction of these newer AOMs into primary care practice has the potential to revolutionize obesity care in conjunction with lifestyle modification. Consequently, primary care providers are positioned to become the main prescribers of AOMs and serve as a reliable source of comprehensive obesity care (14, 15). Yet, what are the possible implications of the introduction of these AOMs for the practice of primary care medicine?

In this paper, we describe early experiences of use of weight loss medications, particularly the newer highly-effective AOMs, among primary care providers, their teams and their patients. We sought to answer the following question: How does the introduction of new AOMs change the practice of primary care medicine with regard to weight management? The answer to this question has important implications regarding the delivery of weight management in primary care, which could alter the weight trajectory of the United States population.

## Materials and methods

### Study context

These findings were gathered as part of a larger study examining a care process for weight management in primary care called PATHWEIGH (not an acronym). PATHWEIGH includes a number of interconnected tools embedded in the Epic^®^ medical record including disappearing help text, care pathway algorithms, connection to UpToDate obesity guidance, billing and coding assistance, mapping of weight and other values progress and more (16). Additionally, the workflow is managed by a new, special visit type called a “weight-prioritized visit” (WPV) where patients have a visit dedicated to weight management (17). PATHWEIGH was studied in a cluster randomized stepped-wedge clinical trial (three cohorts) within one large United States health system and demonstrated effectiveness on patient weight loss and weight loss maintenance (18). Training and consultation support were provided to support implementation. To understand influences on adoption and implementation of PATHWEIGH, extensive qualitative work was undertaken. From this qualitative work, it became apparent that weight loss medications featured prominently as an important factor deserving its own focus.

### Data collection

This study draws on three sources of data: baseline clinician/staff interviews, follow up clinician/staff interviews, and patient interviews (all were collected October 2021 to October of 2023). The primary qualitative data collection method was semi-structured interviews with clinicians (physicians and advanced practice providers) and staff (clinical and administrative) at each of the participating 56 family and general internal medicine clinics receiving PATHWEIGH. The number of interviews conducted by each group are included in the results section. To clarify the lower numbers of staff in follow-up interviews: The follow-up interviews were focused on how weight management was being delivered, perceptions regarding PATHWEIGH as an intervention, and the barriers/facilitators to delivering weight management overall. Since administrative staff were not often not involved in weight management care directly, we invited fewer administrative staff for follow-up interviews. Practice member interviews were conducted at baseline and 1 year into implementation for each cohort. This paper represents interviews conducted at baseline for each cohort (1, 2, and 3) and the 1 year follow-up of cohorts 1 and 2. Purposeful sampling was used to glean the perspectives of representative roles in the practice who were either utilizing PATHWEIGH or providing weight loss assistance in the practice. This resulted in recruitment of 2–5 key clinicians and practice staff members per practice. Semi-structured interviews were also conducted with patients at participating practices. Purposeful sampling was used to recruit patients from a variety of clinic locations, and who either had a WPV or received other weight loss assistance during the study period.

Baseline interviews lasted approximately 45 min and covered the following topics: background of the clinic including patients, staff, and current priorities; perspectives about weight management being conducted in primary care; current weight management practices; interests and goals related to weight management; and plans about starting PATHWEIGH. Follow-up interviews lasted approximately 45 min and included questions about the context of factors related to weight management including patient demand and motivation, use of PATHWEIGH tools, current weight management practices, and barriers and facilitators to providing weight management. Patient interviews lasted approximately 45 min and included questions about the background and demographics of the patient, the length of time the patient had spent at the practice, patient-clinician interactions, current weight management approaches with their clinician, previous attempts at weight loss, and experiences with stigma related to weight management. Interviews were conducted by experienced qualitative researchers from the study team, including two masters-trained professional research services professionals (JW and LC) and two Ph.D.-trained faculty members (CT and JSH). Patient interviews were conducted by one team member (LC). All interviews were digitally recorded and conducted virtually using either Zoom Videoconferencing software or via telephone. Recordings were then professionally transcribed, de-identified, and uploaded to ATLAS.ti qualitative analysis software for data management (versions 9, 22, and 23, ATLAS.ti Scientific Software Development GmbH).

### Data analysis

Analysis occurred in two stages. First, all interviews were coded after each round of data collection using thematic analysis (19). Both *a priori* and emergent codes were identified to categorize interview data. *A priori* codes were identified and defined based on domains from the Practical, Robust Implementation and Sustainability Model (PRISM), the conceptual model informing the larger study (20). Additionally, emergent codes were identified to capture concepts and ideas shared by participants that were not included in the *a priori* codes. The codebook was iteratively developed by all members of the qualitative team (JW, LC, CT, JSH) through multiple rounds of independent coding and reconciliation until sufficient consistency across coders was achieved. The final codebook was then applied to all transcripts (JW, LC, CT, JSH).

During coding, the importance of AOMs emerged as a key factor in the implementation of PATHWEIGH and other weight management approaches. This concept changed over time given that the first interviews were conducted in March of 2021 (3 months before semaglutide first became available in the United States for treatment of chronic weight management), and the latest set in October 2023 (when semaglutide was more widely available). Given the increasingly influential role of AOMs in weight management that our participants described, we then undertook a subsequent analysis of these data to examine the impact of AOMs.

To further explore the role of AOMs in weight management in primary care, we conducted a summative content analysis (21). This approach involves searching for specific words to understand their contextual use and underlying meaning. First, we used the ATLAS.ti “Smart Search Word Search Tool” to locate all instances of target word(s) and their inflected forms: medication, medicine, WeGovy, Ozempic, drugs, GLP, and GLP-1. We then produced an initial query report of all captured instances of the included terms and manually refined the report to ensure that sufficient text surrounding each instance was included. A total of 925 quotations were included in the medication analysis across baseline for cohort 1–3 and follow up for cohorts 1 and 2. Two team members then independently reviewed the reports and wrote analytic memos to summarize content and record emergent themes around the context in which participants described AOMs in weight management. Questions contained in the Box 1 were additionally used to stimulate summaries of the researchers’ analytic memos. Analytic memos were compared and reconciled through iterative team discussion. The “AI summaries” tool in ATLAS.ti was then used to create a summary of the query report. This artificial intelligence summary served to check missingness of major ideas or concepts captured in team members’ analytic memos. To ensure trustworthiness of the findings, two other team members (CT, LC) reviewed the memos and AI summary for accuracy and the themes were refined further through discussions with the entire study team (including three primary care physicians). Finally, to compare the practice member responses to the patients’ responses, a joint display table was created to organize results by thematic areas.

**BOX 1**

How do participants conceptualize obesity and how does that relate to whether they endorse and/or use these medications?

• Medical condition needing lifetime management or lifestyle issues that should not require medication

What do people think about the medications themselves? Are they effective? Are they concerned about side effects or adverse issues?

What are the challenges to using the medications?

Summarize the following factors and what the challenges are and why they are challenges. What might be recommended?

• Availability of medications
• Insurance coverage (whether covered or not and thechallenge of figuring out if it is covered or not)
• Patient response (i.e. how much they want it anddemand getting it)

What are the benefits of having these new medications?

Summarize the following factors and why each seems to be an issue.

Patient response (how it is actually working for some patients)
Practice response (actually having something that can be prescribed that works)

How do the providers actually use medications for weight loss? (all types not just the new ones)

Consider:

• When in the visit, with which patients, how they do it, anything they say to patients.
• How has the availability of new medications changed the conversation about primary care delivering weight loss services if at all?

What else emerges from the interviews?

## Results

See Table 1 for practice personnel types and practice locations. Table 2 displays patient participant demographic information and Table 3 shows the joint display table by thematic areas with representative quotations.

**TABLE 1:** Practice participant demographics.

Practice personnel role type or practice location	Baseline				1 year follow-up		
	Cohort 1	Cohort 2	Cohort 3	Total	Cohort 1	Cohort 2	Total
**Personnel role type:**							
Clinicians	28	26	18	72	21	10	31
Clinical staff	31	16	2	49	10	7	17
Administrative staff	24	15	5	44	4	3	7
Total	83	57	25	165	35	20	55
**Practice location:**							
North	6	4	3	13	6	4	10
Metro	10	9	9	28	9	9	18
South	4	4	3	11	3	4	7
Total	20	17	15	52	18	17	35

**TABLE 2:** Patient participant demographics across all cohorts.

Characteristic	Variable	*N*
Gender identity	Male	3
	Female	16
Insurance status	Government (Medicare, Medicaid, VA)	8
	Commercial/private	11
Age range	Under 40	7
	40–65	8
	Over 65	4

**TABLE 3:** Comparison of practice member and patient thematic findings.

Practice member themes	Patient themes	Quotations	Comparison
The new medications are a game changer. Finally, something effective is available to offer to patients	These medications have changed my life	Care manager: “*Well, I know certainly now that some of the medications are available that were not available when the program first started. I think that that is going to be helpful because it gives the providers, it makes them feel like they have more tools in their tool belt to help patients.*” Patient: “*Really if I stopped losing weight today and I was just going to be 205 pounds for the rest of my life, I would still stay on this medication for the rest of my life because I’m just so much better in every way. Not only just the weight and just so much better, but I would still stay on it just to feel this freedom from being obsessed with food*….”	Both practice members and patients recognize the impact on weight loss success for patients, not just in terms of weight loss but effects on freedom from food obsession, ability to move and also reductions and improvement in other health conditions
The new medications come with significant burden for everyone	Accessing these medications is frustrating – hard to get, hard to pay for	Physician: “…*The message that comes across*…*is just prescribe these medicines and we’re going to get some real progress, but then insurance won’t cover this. And the patient feels kind of mixed messages because the provider said we’re going to get you on these shots and then we can’t get them. They’re not affordable. So, there’s a little bit of frustration there.*” *Clinic Manager: “Not that I have heard of in terms of the actual weight prioritized visit itself, but the struggle is for patients being able to get the prescriptions filled and with medication shortages and then patients not understanding that a lot of those meds require prior authorization*…*It takes more time adding that to our prior auth team having to obtain prior auths because now we’re prescribing those medications more.*” Patient: “*I called my insurance company yesterday to see if they would cover the diabetes dietitian, and they said no*…*so I sent Dr. [NAME] a message and said – well, first off, “I can’t go to the dietician that you recommended. And secondly, it looks like there’s a chance the [medication] was denied,” that insurance won’t cover it. And so we’re waiting to find out that for sure*…*I feel like we keep hitting these roadblocks, and I’m not really getting anywhere.*”	Both practice members and patients were frustrated by the numerous burdens of obtaining these medications for patients
Not everyone is on board with using medications, or using them for all circumstances and the reasons are varied	Medications should only be used under certain circumstances	RN: “*We have one provider who will not, I won’t say will not. She really tries not to use the medications. She doesn’t like the concept of what she calls the magic pill*… *She wants them to kind of put in the work per se. So, she is heavy on that route and then one of our other docs is heavy on medications.*” *PharmD: “We have also seen patients not tolerate these medications as well, so having side effects like nausea, had patients who’ve ended up in the ED with vomiting, so they’re not without side effects. And so that could potentially be a barrier too. Although I really think it’s more cost driven at this point.*” Patient: “*(Patients should) have that experience with trying different avenues, not just the provider saying, okay, here, you want to lose some weight, here’s this prescription. (Providers should) make sure that the patient is willing to put in the work with the exercise and the work with still eating healthy and eating all the correct things*… *prescription medication to aid in weight loss is exactly that – it’s just an aid to help. It’s not a fix, it’s not going to do everything for you. So I think that just the provider’s having that holistic look and making sure that the patients are going to be consistent and that’s not that quick magic pill*…*(patients should have) done a lot of other things first.*”	Both groups had instances where medications were thought to not be the treatment at all or for individual patients or circumstances, however, this varied by the patient and the provider involved
Changing conceptualization of obesity as a chronic disease	Patients still view weight management as “a me problem” and offering medications made them feel supported and not shamed for their weight	Physician: “*We’re starting to see obesity as a chronic disease process and we really have a steep learning curve to help people overcome it. And we’re learning things. I don’t promise miracles, but I try to generate some enthusiasm for working on it, for coming back.*” Physician: “*I think it can be treated much like an addiction and I think it can lead to other medical complications. However, I think we also have to be sensitive that it doesn’t always kind of in the same way that people with high cholesterol don’t always get a heart attack. It certainly could.*” Patient: “*(I needed) understanding that I have put in an effort that I’m not just saying “Hey, help me” in like a lazy way. But more so as like a I’ve done (a lot), like I’m seeking help from desperation.*” *Patient: “We talk about my mood. I have a lot of postpartum depression and just how that can affect overall wanting to exercise and eat healthy. She talks about all that, like how it’s not just the eating that can affect all this. It’s mood; it’s my medications that I’m on outside of the phentermine that play a role in my body.*”	Although similar in theme, providers and teams relayed their changing conceptualization more around considering obesity as a chronic disease, whereas patients shared more about their experience of their provider considering the broader picture and understanding more factors than just individual discipline
Changing care patterns of weight management in the practice of primary care	Absent	RN: “*The (comments on providing obesity care) all been very positive. Everybody that I’ve talked to said, wow, that’s amazing. Yeah, I would love to do that.*” Physician Assistant: “*My impression was, thank goodness. We’re going to have a structured tool for (doing obesity care)*… *I was talking to Dr. (NAME)*… *and there were two things that I felt were really missing from my ability to help people. And one was a psychologist because I found when you really dig into people’s weight patterns and eating patterns, there is a lot of psychological overlay that is very difficult to address in a family practice setting. And then the other thing was (patients) often asked to see a dietitian. And I know we have some availability to do that, but a lot of these people don’t have diabetes and they don’t have comorbidities where their payer would want to do that. And I guess maybe I’m remiss on seeing how much that’s available for people whose only problem is 35 years-old with a BMI of 40 and no other health problems.*”	Patients did not vocalize much on this theme although there was some recognition of wanting obesity care as part of their regular health care in primary care, whereas providers and teams saw this a changing from essentially not providing this care to now providing it and having a means to do so

### Practice member themes

#### Theme 1: the new medications are transformative, “a game changer.” Finally, something effective is available to offer to patients

• Past options included mostly phentermine, which is a controlled substance. Many providers did not feel comfortable prescribing it for long periods due to the potential cardiac side effects. Although this medication is not as effective as other weight loss medications, it is still prescribed due to its low cost and accessibility.
• There were reports of some amazing results for patients, including substantial weight loss and high satisfaction with the results of using AOMs among those for whom the treatments were successful.
• Patients who come to primary care had frequently tried other methods and were looking for something new. These new medications have revitalized interest in weight management in a new way.

#### Theme 2: the new medications come with significant burden for everyone

• Supply issues were widespread for semaglutide and tirzepatide regardless of their branded indication.
• Many insurances did not cover the cost of the medications, and even if they did, patients could be charged significant co-payments. Out-of-pocket costs were expensive, and while compounded versions became more widely available at lower prices, the use of compounding pharmacies raised concerns for some providers due to safety concerns. The issues around cost contributed to disparities in access to these medications, particularly for those with lower incomes or living in rural areas.
• There was uncertainty regarding insurance policies and procedures, or “not knowing the rules of the game” and thus coverage, access, and costs. This opacity led to many providers engaging in trial-and-error strategies, which felt like “flying blind.” Figuring out these complex and varied details consumed significant staff time, which was perceived as “wasted time.” This situation was extraordinarily challenging and frustrating for patients and providers alike, with some physicians giving up on prescribing these medications due to the substantial time and effort required to manage this complexity.
• Practices reported that patient demand can add to the burden on their clinic. Patients requested a specific medication, sometimes insistently or demandingly. Some providers reported having some patients who are unwilling to make necessary lifestyle changes, only wanting the “magic pill,” which was a turnoff to providers. Some patients left their providers over this conflict.

#### Theme 3: not everyone supported the use of AOMs, or using them for all circumstances; the reasons for this are varied

• There were some concerns about putting patients on medications due to reported side effects and problems (e.g., emergency room visits for fainting or gastroparesis), as well as the implications of prescribing a lifetime medication (e.g., the risk of regaining weight after discontinuation). This is in contrast to attitudes toward other chronic, lifelong medications such as antihypertensives or hypoglycemics.
• Providers reported that some patients were averse to take medications or saw it as “cheating.” Others were reluctant to perform self-injection. However, many were desperate for help and reported being willing to do almost anything for weight loss.

#### Theme 4: changing conceptualization of obesity as a chronic disease

• Many providers shifted from viewing obesity as a “lifestyle failing” to recognizing it as a chronic disease requiring treatment. However, this perspective was not universal; some still believed that because they (or some patients) could manage their weight through lifestyle changes, everyone else should be able to do the same. There appeared to be a trend among older physicians, who appeared to hold the belief that people need to “do the hard work.”
• Accompanying this conceptualization of obesity as a chronic disease were sentiments of normalizing the medicalization of weight loss and maintenance. Like any other chronic disease, it was considered acceptable to take medications if needed. However, opinions about and comfort with using medications off-label differed, even among those who are comfortable doing so for other conditions.
• Although many wanted to try lifestyle changes first with their patients, some felt taking this approach made the medications a “life preserver,” instead of acknowledging that the problem has gone on a long time. It was acknowledged that patients still do need lifestyle support even when on medications (analogous to bariatric surgery), and many clinicians did not feel comfortable just prescribing medications without additional support.

#### Theme 5: changing care patterns of weight management in primary care

• Many reported an increase in visits focused on weight management, not just because of PATHWEIGH but because there was now an effective solution (AOMs). Both patients and providers were more willing to engage in weight management in primary care because of viable options beyond the typical advice to “eat less and exercise more.”
• There was a noted increase in visits for weight management in general and follow-up discussions centered around prescribing AOMs.
• Having more medication options available gave providers additional tools to help patients, especially for those who reported having tried everything else. Patients who came to their physician for weight management often sought medication because it was something they could actively do.
• The availability of these medications sparked renewed interest among providers in learning more about weight management and how to help their individual patients, re-engaging them in an important aspect of primary care.
• Regardless of medication use, respondents felt that better access to diet, exercise, and other resources is needed. Behavioral health was not widely utilized for weight loss, even in those practices with embedded behavioral health specialists trained in behavior change. Some behavioralists were not comfortable with the topic, others were too busy, while still others perceived their role as more oriented to issues such as mood disorders rather than behavior change. There was also limited access to registered dietitian nutritionists or other sources for quality diet advice.

### Patient themes

#### Theme 1: “these medications have changed my life”

• Patients reported significant weight loss with the introduction of AOMs. Many of them had never previously tried a weight loss medication and described this as “life changing,” enabling them to live life more fully, with reduced stress, increased comfort, and the ability to overcome life circumstances that made previous attempts at weight loss challenging.
• Many patients found the traditional advice to “eat less and exercise more” to be impractical. With the aid of medications, they were better able to manage their diet and exercise along with family responsibilities.
• Patients experienced a sense of liberation from constant preoccupation with their weight, obsessions with food and emotional eating patterns. For example, one patient reported that she could attend a conference and enjoy the holidays without meticulously tracking her food consumption.
• Many patients reported enhanced mobility and the ability to discontinue other medications for weight-related conditions, such as antihypertensives.

#### Theme 2: challenges in accessing and affording medications

• Patients overwhelmingly reported significant barriers when attempting to access weight loss medications. Obtaining coverage or reimbursement for these medications often felt like a moving target, with many experiencing coverage denials or having to navigate numerous obstacles to secure approval and receive their medications.
• Patients described encountering “a lot of hoops you’ve got to jump through for insurance to cover (the medication).” The price of injectable AOMs posed financial and psychological barriers for some: “$150 out of pocket…is a lot of money.” For many patients, the support of their primary care provider in navigating this process was crucial, as was their own persistence and assertiveness in dealing with insurance companies.
• Patients also reported challenges accessing medication at the pharmacy, including delays with refills that necessitated making refill requests earlier and more frequently. The availability of medications also impacted some patients’ treatment; one patient resorted to taking phentermine because semaglutide was unavailable.

#### Theme 3: medications should only be used under certain circumstances

• Many patients felt that AOMs should not be offered as initial therapy but rather as a welcome middle ground between traditional “eat less and exercise more” advice and bariatric surgery. They viewed medications as a “springboard” to be considered only after multiple attempts to manage weight with behavior changes had been unsuccessful.
• While some patients believed that medications should only be offered after behavioral approaches, they also reported that they should be tried before resorting to invasive options like bariatric surgery. Among those patients who had been offered surgery as the only viable option beyond lifestyle changes, only one was interested in this option. Nearly all felt that surgery was proposed too soon - before they had exhausted all other options. For these patients, bariatric surgery was seen as like a serious procedure that should be reserved as a last resort.

#### Theme 4: patients still view weight management as “a me problem,” and offering medication provided relief from self-blame and shame, providing support and reducing stigma

• Numerous patients reported that this was the first time that medications for weight loss were offered to them. One patient reported that she had been interested in medications but never asked, assuming that she would be turned down and only offered the same basic advice she had heard before. Similarly, other patients expressed a need for acknowledgment of their efforts before they would consider medications.
• Additionally, it was important for patients to differentiate between official AOMs and other “drugs,” including amphetamines, supplements, and unregulated over-the-counter or compounded options. Patients valued knowing that the weight loss medications were FDA-approved (unlike OTC drugs) and that clinicians had other patients who benefited from them. They appreciated information about the safety and effectiveness of these options.
• Patients reported that the manner in which the medications were offered was crucial. They particularly valued discussions about mediations within the broader context of their overall health. Patients appreciated when their providers took a holistic view, acknowledging their prior behaviorally-based attempts to lose weight, and communicating the biological underpinnings of their weight management experiences (e.g., hormones, insulin resistance). Patients recognized the value of addressing weight in primary care versus through specialists for this reason, noting that specialists often take a “piecemeal approach” by “only looking at that one small piece of it.”

### Cross-cutting findings

A comparison of the themes from practices members and patients indicates substantial alignment of many themes from both groups. This substantial agreement is highlighted in Table 3. This analysis also revealed two unique concepts regarding the use of AOMs for weight management in primary care.

#### Concept 1: the medication cascade of events

**FIGURE 1:**
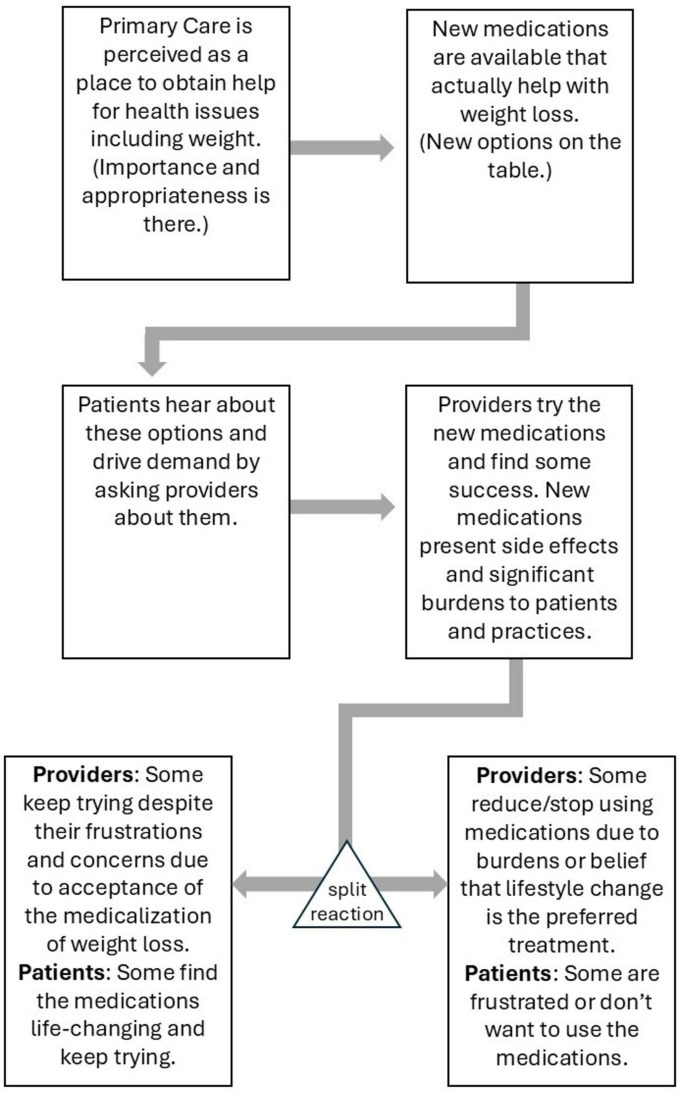
Medication cascade of events.

Collectively, these findings illustrate an intricate cascade of events that resembled a roller coaster ride for all involved. Interviewees often made use of emotional language, frequently referring to the process as “frustrating.” This cascade is depicted in Figure 1, outlining the series of events.

#### Concept 2: the right fit conundrum

Related to the medication cascade is what we call the “right fit conundrum,” which illustrates that successfully prescribing medications for weight loss in primary care is dependent on a number of factors that must all align and is challenging to accomplish. Findings from interviews showed that primary care providers had an opportunity to engage in conversations about weight. It appeared that a perfect cascade of events had to occur in order for weight loss medication to be prescribed in a successful way. In other words, getting the right thing to the right patient at the right time.

Key factors related to the “right fit conundrum” are:

• Right patient: those who have tried other things and needed new solutions and were willing and able to do the work needed (i.e., the needed lifestyle changes); some patients do not want medications.
• Right provider: those who had a mental model of understanding obesity as a disease and were willing to learn and work with patients on weight loss treatments. Those who were comfortable with using medications and providing support to patients as part of their care.
• Right resources: having the insurance coverage and supply of medications for the long-term to cover the extended use of the medication, the support in terms of regular follow-up and behavioral and nutrition support, for the physicians to have the support to normalize and know what to do regarding patient instructions related to diet change.
• Right treatment: the desire to take medication was patient-dependent, however, there were external factors related to the supply of medication which can lead to equitable access issues for patients who would benefit from treatment.
• Right fit between clinician and patient (therapeutic partnership): there were some providers who are not ready/willing to engage due to lack of time or support or tools or philosophy. The data also showed that a mismatch between a patient’s desires and a provider’s willingness to prescribe certain medications was a barrier for patients.

## Discussion

The arrival of new highly effective incretin injectable weight loss medications appears to significantly influence weight management in primary care settings (22). These changes have both positive aspects (finally, something that works!) to the negative (what a hassle!). This study clarified that patients and primary care teams share similar views on those benefits and burdens. In particular, this paper highlights the cascade of events necessary for the successful and effective use of these medications for weight loss in primary care.

While many studies are emerging on the effectiveness of AOMs and recommendations about their use (22–25), there is little research on patients’ perceptions or how these medications may be changing medical practice (26). Lacking in the discussion of these AOMs are the social, cultural and clinical contexts in which decisions are made about how obesity is managed. A lack of education about and use of obesity treatments still persists (27), with many clinicians feeling ill-prepared by their training and unsupported by the health care system to address the lifestyle aspects of managing obesity, such that obesity may not be perceived as something primarily within their domain to address (28). Societally, many clinicians and others believe that obesity exists as a lifestyle management failing, such that individuals with obesity need to simply have more discipline to institute different diet and exercise patterns (29). Thus, there is a diminishing but still present stigma regarding weight and use of AOMs (30, 31). However, the conceptualization of obesity as a chronic disease has increased in recent years, and thus as a condition worthy of medical treatment rather than a personal failing has become increasingly apparent (32, 33). The implications for this changing conceptualization may result in more clinicians being willing to prescribe AOMs as a treatment approach.

In the United States, media attention on AOMs has been impressive and has influenced patient demand. It is reported that these are now among the most expensive medications available, placing significant financial strain on payers (25). Their emergence has also highlighted the stark disparity between those who have access to these medications and those who do not, such as Medicaid recipients and the un- and underinsured. Furthermore, clinicians and their teams have struggled to obtain these medications for their patients in the face of an increasingly burdensome and opaque process involving not only insurance authorization but also production shortages, local pharmacy inventory, and the potential risks of compounded products. They have had to prescribe these medications without knowing if it is covered, whether or where it can be obtained, if that pharmacy participates in the patient’s benefit management program, or whether the patient can afford it. While they may be comfortable prescribing these medications for diabetes, they may have reservations about newly emergent side effects, long-term safety, and the possibility of lifelong reliance on a medication for what they may perceive as a lifestyle issue (34–36). Deprescribing of AOMs is a topic currently under study, with the trial results demonstrating largely that removal of the AOMs causes significant weight regain and many studies underway examining different options for successfully transitioning off the AOMs (37, 38). Acceptance of chronic medications for obesity may evolve as the aforementioned conceptualization of obesity as a chronic disease develops. Thus, treatment may be more analogous to hypertension or diabetes, where lifestyle changes assist, but lifetime medication is typically required to achieve desired outcomes.

There are several key messages from this analysis. First, weight is a complex issue with significant variation in the attitudes and approach of patients and providers. However, the conceptualization of weight and weight management appears to be shifting. These results suggest the importance of developing personalized approaches based on patients’ needs, interests, attitudes, experiences, and available resources. Primary care is well-positioned to provide this care, given its core values of care that is comprehensive, coordinated, collaborative and person-centered, encapsulated by the phrase “*Meet them where they are.*” Second, numerous factors must align for patients and the primary care team supporting them to achieve successful outcomes with the newer weight loss medications – finding the “right fit alignment” can help to address challenges and alleviate frustrations that may arise during this process.

Limitations of this work include the qualitative nature of this study, which was conducted during an ongoing clinical trial. Here we report the perceptions and experiences of a large number of practice members as they encountered utilization of these medicines over time. This clinical trial is being conducted in one western state health system with patients, although diverse in geographic location, age, and gender, are specific to that setting and may not be representative. Data collection and analysis for this study corresponded with the emergence of these medications, and with shifting access, availability and insurance coverage for them; this included a period when COVID-19 restrictions were in place. Lastly, these data were collected as part of a study designed to provide support and assistance to primary care practice clinicians and staff to utilize specific visit types and tools within clinical practice, which may not be present in other settings. However, all practices within the health system were enrolled to receive this intervention, suggesting there is no inherent bias toward more interest and willingness to participate in weight loss care than other typical primary care settings.

In summary, the introduction of highly effective weight loss medications may invigorate efforts to integrate weight management into primary care, but the long-term implications of this shift are still unknown. Further exploration of the effects on patients, providers, and care paradigms is warranted. Managing the obesity crisis will require further education and research into how best to integrate into primary care comprehensive weight management, including both effective patient support as well as new and emerging therapies.

## Data Availability

The raw data supporting the conclusions of this article will be made available by the authors, without undue reservation.
